# Eye diseases during pregnancy: a study with the medical data warehouse in the eye clinic of the Ludwig-Maximilians-Universität München in Munich in Germany

**DOI:** 10.31744/einstein_journal/2022AO6613

**Published:** 2022-04-28

**Authors:** Thiago Gonçalves dos Santos Martins, Paulo Schor, Luís Guilherme Arneiro Mendes, Andreas Anschütz, Rufino Silva

**Affiliations:** 1 Ludwig-Maximilians-Universität München Munich Germany Ludwig-Maximilians-Universität München, Munich, Germany.; 2 Universidade Federal de São Paulo São Paulo SP Brazil Universidade Federal de São Paulo, São Paulo, SP, Brazil.; 3 Centro Hospitalar e Universitário de Coimbra Coimbra Portugal Centro Hospitalar e Universitário de Coimbra, Coimbra, Portugal.

**Keywords:** Pregnancy complications, Data warehousing, Eye diseases/complications, Big data

## Abstract

**Objective:**

To analyze the most common ophthalmologic disorders in pregnant women seen in a hospital in Munich in Germany using a big data analysis system, as well as to compare the results obtained with those from other epidemiological studies that used different data acquisition methods.

**Methods:**

We retrospectively analyzed electronic health records of pregnant women who were seen at the ophthalmology department from 2003 to 2019 at the Ludwig-Maximilians-Universität München hospital. The main complaints that led to ophthalmic consultations during this period were evaluated, and also the variation in intraocular pressure of patients throughout gestational trimesters by analyzing data from the data warehouse system.

**Results:**

A total of 27,326 electronic health records were analyzed. Of participants, 149 (0.54%) required eye care during pregnancy. Their mean intraocular pressure was 17mmHg in the first trimester, 12mmHg in the second trimester, and 14mmHg in the third trimester. The most prevalent findings were dry eye (29.3%) and conjunctivitis (16%), and ametropia (16%). The most common posterior segment problem was diabetic retinopathy (4.6%). The lower mean intraocular pressure in the second and third trimester found in our study is in accordance with other studies that used other method for data acquisition.

**Conclusion:**

The most common ophthalmic conditions found in this study population were dry eye, conjunctivitis, and ametropia. The use of data warehouse proved to be useful for acquiring and analyzing data from many patients. This study results are comparable with other studies in published literature that adopted different methodology.

## INTRODUCTION

About 90% of the planet’s data was generated in the last 2 years. Google estimates that mankind has created about 300 exabytes of data in the last 5 years. Medicine has produced a great amount of data due to the use of electronic health records (EHRs). This significant amount of data exceeds human capacity and requires the development of algorithms to analyze the information.^(1)^ One way to analyze this large amount of data is to use a data warehouse, which is a system that gathers information from different sources. Depending on the information required to develop a study, different methodologies can be adopted. The data warehouse is built to hold a large amount of data and enables its rapid analysis, which creates opportunities to develop more studies with large samples in less time and at a low cost. Epidemiological studies with large samples are important to establish clinical guidelines.^(2-5)^ Studies based on the information from large electronic databases can be developed using the data warehouse, which extracts important information such as ophthalmic disease classification and patients’ intraocular pressure. The ability to collect and store data has far surpassed our ability to analyze, summarize and extract knowledge from them. The data warehouse is useful for processing a large amount of medical data into valid and reliable information that could be used to assist the decision making process.^(4,5)^

During pregnancy there are physiological changes that could cause pathological eye conditions or interfere with pre-existing ones. Some of these changes are diabetic retinopathy, central serous chorioretinopathy, preeclampsia and eclampsia, uveitis, idiopathic intracranial hypertension, and pituitary tumors. Most ophthalmic manifestations are due to the metabolic, hormonal and immunological changes that occur during pregnancy. Most of changes do not cause visual sequelae.^(6,7)^

## OBJECTIVE

To analyze the most common ophthalmic manifestations in pregnant women seen in a hospital in Munich in Germany by using a big data analysis system, and then compare the results with those from other epidemiological studies that used other data acquisition methods.

## METHODS

### Study setting and design

This was a cross-sectional study that analyzed the health records of women who gave birth from 2003 to 2019 and were seen during pregnancy at the Department of Ophthalmology at a single tertiary referral center in Germany (University Eye Hospital, Ludwig-Maximilians-Universität München, Munich, Germany). All data warehouse queries were approved by the local data protection officer. Written informed consent for the retrospective use of anonymized data was provided by all patients included in this study. All procedures performed in studies involving human participants were in accordance with the ethical standards of the University Hospital Munich, Ludwig-Maximilians-Universität München, Germany. This study follows the 1964 Helsinki declaration and its later amendments or comparable ethical standards.

### Data source and patient characteristics

We retrospectively reviewed the EHRs of all women who gave birth from 2003 to 2019 at the Ludwig-Maximilians-Universität München of Germany hospital. All patients requiring eye care in the 9-months period prior to delivery were included for analysis, whereas those with incomplete data or those without ocular manifestations were excluded. The chief complains and intraocular pressures in the three trimesters were collected. Data from the EHRs was extracted, transformed into consistent data, and then loaded into the data warehouse. All clinical data used for this study was queried from the data warehouse. This warehouse was established in 2013, and since then has been collecting information from the EHRs and diagnostic devices from more than 330,000 patients. It contains clinical findings from the patients’ EHR and results from previous studies.^(3,4)^

Information was extracted from the data warehouse according to the following: firstly, all patients’ records containing the International Classification of Diseases tenth revision (ICD-10) code of delivery were identified. From this group, patients that require eye care in the 9-months period prior to delivery were selected. Exclusion criteria were incomplete data from the medical record. All the participants had undergone a comprehensive ophthalmological examination, which included medical history, visual acuity, refraction, intraocular pressure measurements with Goldmann applanation tonometry, and a slit-lamp examination.

### Statistical methods

All statistical analyses were performed by using the programming language “R” (R Foundation for Statistical Computing, Vienna, Austria). The relevant data for the study was retrieved from the data warehouse with Sequence Query Language Statements (SQL). The boxplot was calculated with the R plot function ggplot with two variables, intra ocular pressure of the patients and trimester. The box starts at the 25^th^ percentile and ends at the 75^th^ percentile of the patients. The black line in the box is the 50^th^ percentile and the end of the lines are marking the lowest and the highest level of the measurements.

## RESULTS

**Figure 1:**
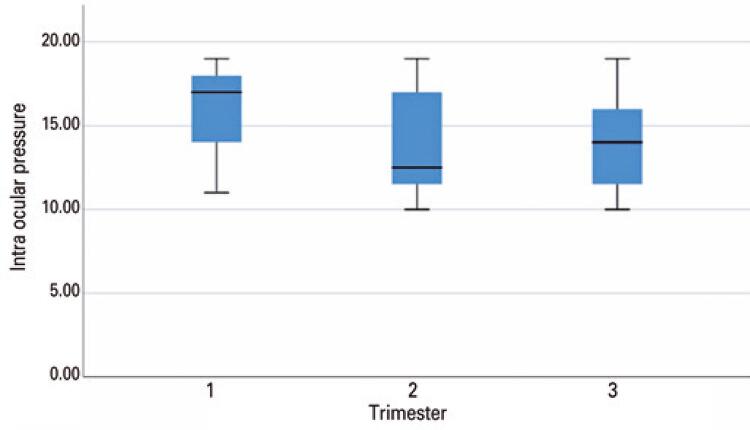
Individuals mean intraocular pressure was 17mmHg in the first trimester, 12mmHg in the second trimester, and 14mmHg in the third trimester

Of 27,326 women who gave birth from 2003 to 2019, only 149 required eye care during pregnancy (0.54%). The patients’ age ranged from 17 to 45 years old, with a mean of 32 years old. Their mean intraocular pressure was 17mmHg in the first trimester, 12mmHg in the second trimester, and 14mmHg in the third trimester (Figure 1). The most prevalent diseases were those affecting the ocular surface and ametropia: dry eye (29.3%), conjunctivitis (16%), and ametropia (16%). The main alteration in the posterior pole was diabetic retinopathy (4.6%) (Table 1).

**Table 1:** Most common chief complaints from pregnant women at the Ludwig-Maximilians-Universität München hospital from 2003 to 2019. The percentile of diseases diagnosed at the pregnant patients was gained with Sequence Query Language Statements in the data warehouse

Ophthalmological manifestations	(%)
Dry eye	29.3
Refractive error	16
Conjunctivitis	16
Hordeolum/chalazion	9.7
Subconjunctival hemorrhage	6,2
Diabetic retinopathy	4.6
Other causes	18.2

**Figure 2:**
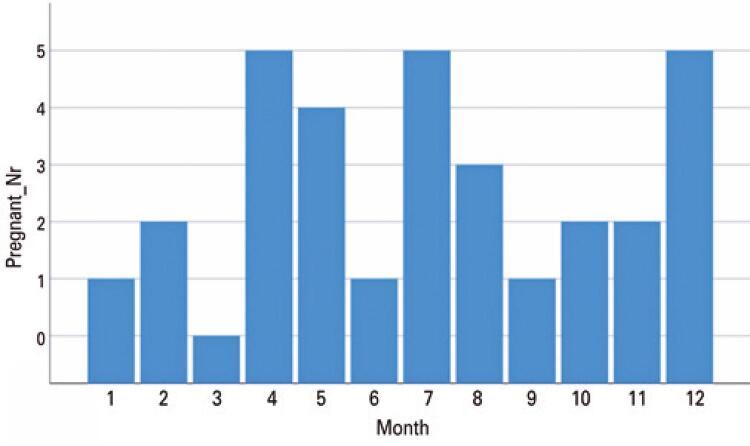
The numbers of all pregnant women requiring eye care were compared (149) with the total number of patients requiring eye care in the same period (27,2873)

Cases of conjunctivitis were also documented in the general population seen at the same hospital from 2003 to 2019. The numbers of all pregnant women requiring eye care were compared (149) with the total number of patients requiring eye care in the same period (27,2873) (Figure 2). The general population (27,2873) had a higher prevalence of conjunctivitis from April to June (spring in Germany) while the pregnant women exhibited higher peaks in April, May, July, and December.

**Figure 3:**
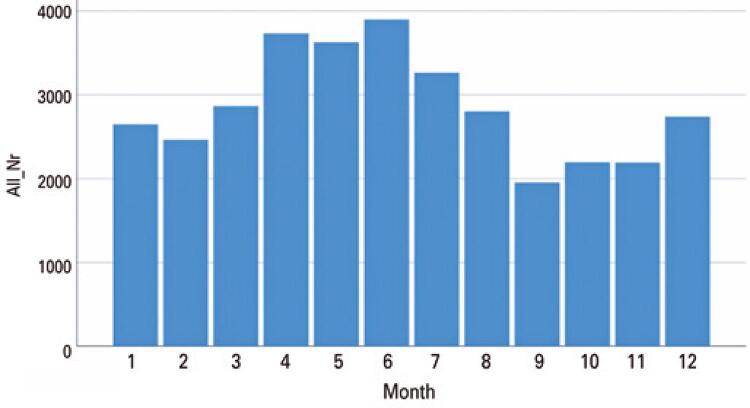
Seasonality of conjunctivitis (allergic and infectious) cases in the general population with the pregnant population that were seen in clinic from 2003-2019

We also compared the seasonality of conjunctivitis (allergic and infectious) cases in the general population with the pregnant population that were seen in clinical settings from 2003-2019 (Figure 3). The general population (27,2873) had a higher prevalence of conjunctivitis from April to June (Spring in Germany), which reflects an increase in allergic related conditions. The group of pregnant women exhibited higher peaks of conjunctivitis in April, May, July, and December.

### Comparison with other studies

Our study showed similar results when compared with other epidemiological studies that used different methods of acquiring the data. The lower mean intraocular pressure in the second and third trimester found in our study was in accordance with other studies described in the table 2.^(8-11)^ The intraocular pressure may decrease by an average of 2mmHg to 3mmHg in the second trimester of pregnancy and tends to return to the baseline two months after the delivery.^(8,12)^

**Table 2:** Comparison of studies previously performed

Study	Country	Study design	Number of patients	IOP
Efe et al.,^(9)^	Turkey	Prospective cohort	25 patients	Lower in 2^nd^ and 3^rd^ trimesters
Green et al.,^(10)^	United Kingdom	Prospective cohort	7 patients	Lower in 2^nd^ and 3^rd^ trimesters
Qureshi^(11)^	Pakistan	Prospective cohort	36 patients	Lower in 2^nd^ and 3^rd^ trimesters
Ebeigbe et al.,^(12)^	Nigeria	Prospective cohort	117 patients	Lower in 2^nd^ and 3^rd^ trimesters
Martins et al.,	Germany	Cross-sectional descriptive study (data warehouse)	149 patients	Lower in 2^nd^ and 3^rd^ trimesters

IOP: intraocular pressure.

Other studies have also pointed out a different seasonal presentation of some diseases in pregnant women. For example, a survey conducted in Denmark revealed a seasonal variation in hypertensive disease of pregnancy, probably related to vitamin D levels fluctuation.^(13)^ The different seasonal presentation of conjunctivitis between pregnant women and general population that we noticed in our study could be better elucidated in future prospective cohorts.

## DISCUSSION

According to the results, only 149 pregnant women (0.54%) sought ophthalmic care at hospital where the study was conducted. This number may be underestimated, since some of the pregnant women may have been seen at other hospitals. The mean age of these 149 pregnant women was 32 years old. The mean age of women at the birth of their first child in Germany is 31,2 years old and 30.9 years in the European Union, respectively.^(14)^ As most ophthalmic manifestations that occur during pregnancy do not inflict major changes in patients’ eye health and these numbers may be underreported. Their mean intraocular pressure was 17mmHg in the first trimester, 12mmHg in the second trimester, and 14mmHg in the third trimester. The intraocular pressure of all patients was not measured in all trimesters. The mean intraocular pressure measured in each trimester was calculated from those who had this information on file. Only 1.3% of patients had a diagnosis of glaucoma. Thus, the prevalence of glaucoma in pregnant women at hospital is lower than that of the adult population worldwide (2%), and lower than the adult population older than 40 years old in Europe (2,4%).^(15,16)^

The intraocular pressure may decrease during pregnancy and the rate of aqueous humor formation has been shown to remain constant during pregnancy. However, there is an increase of the flow, which may cause a decrease in intraocular pressure. There is also a decrease in episcleral venous pressure. It has been reported that this decrease might be due to a decrease in general peripheral vascular resistance during pregnancy and might contribute to a further decrease in intraocular pressure.^(8-16)^

However, another theory postulates that the intraocular pressure does not actually decrease, but the decrease of corneoescleral rigidity during pregnancy would lead to false low intraocular pressure measurements during applanation tonometry. During the second and third trimesters of pregnancy, a paradoxical state occurs in which the cornea is thickened as a result of increased corneal hydration, and intraocular pressure measurements tend to be lower. Thus, decreased intraocular pressure values during pregnancy may partly reflect decreased corneal rigidity rather than true intraocular pressure decreases.^(10-18)^

The most prevalent diseases were those affecting the ocular surface and ametropia: dry eye (29.3%), conjunctivitis (16%), and ametropia (16%). During pregnancy, the cornea’s sensitivity is reduced, and its thickness is increased. These physiological changes can worsen dry eye symptoms and increase contact lens intolerance. The cornea tends to retain more water during pregnancy, and this could increase the myopia shift. Thus, refractive surgeries should be avoided during this period. There is also decrease in accommodation and in the production of tears, facilitating dry eye and infections. According to data from published literature, up to 10% of pregnant women may develop conjunctival hemorrhage in the postpartum period.^(17-23)^A study showed that 6.2% of pregnant women that sought eye care were treated for subconjunctival hemorrhage, which usually evolved without sequelae after 2 to 3 weeks.

The group of pregnant women exhibited higher peaks of conjunctivitis in April, May, July, and December. This difference may be explained by the immunological changes during gestation. High cortisol levels are associated with immunosuppressive mechanisms, as well as changes in the production and composition of the tear film, which, in addition to worsening dry eye symptoms may increase the risk of conjunctival infections.

Both pregnancy and atopy are characterized by a shift from cellular (T-helper cell type 1-dominated) towards humoral immune responses (T-helper cell type 2-dominated). Pregnancy promotes this immunological shift in order to prevent fetus rejection while maintaining a defense mechanism against infections.^(18)^

Pregnant women may have ptosis due to the fluid retention caused by hormonal changes that affect eyelid aponeurosis. Ptosis usually improves after delivery.^(19)^ During the study, only 0.6% of the pregnant women were treated for eyelid changes.

Symptoms of preeclampsia and eclampsia may occur in 5% of pregnancies. Symptoms include hypertension, proteinuria, scotomas, diplopia, hemorrhages and vascular changes in the retina, papilledema, ischemic optic neuropathy, and serous retinal detachment.^(24)^ During the research period, 1.3% of pregnant women had serous retinal detachment and 1.3% of them had papilledema.

During the study period, 1.3% of pregnant women presented retinal venous occlusion. There is a hypercoagulability state that results from pregnancy-related changes that can lead to occlusion of the central retinal branch and vein.^(25)^

Diabetic retinopathy was the main cause of ophthalmic follow-up in 4.6%. Pregnancy may be related to increased progression of diabetic retinopathy, which usually improves after the third trimester and delivery. Pathophysiology may be related to decreased blood flow, which worsens retinal ischemia and accelerates the progression of diabetic retinopathy. The treatment is the same as in any other patient. Macular edema can be followed up conservatively because it usually regresses after the delivery. There are no contraindications for laser use, but the use of intravitreal antivascular endothelial growth factor (anti-VEGF) injections should be done with caution, as they may interfere with placental vascularization.^(26-28)^

### Strengths and limitations of the study

Strengths of the present study include, to the best of our knowledge, the fact that this is the first study that used data warehouse to analyze pregnant women seen in the eye clinic between 2003 and 2019. Some limitations of this study should be addressed. Such as, the data reflects the prevalence of ophthalmological manifestations in patients seen at a hospital in Germany, which may not be generalized to different settings. The retrospective design relies on database records. The high prevalence of dry eye obtained may be due to its high prevalence in the general population. Many ophthalmologic manifestations are asymptomatic and may have a higher prevalence than that presented in the study. The number of pregnant women with eye complaints may be underestimated because they may have been seen at other hospitals. Further studies including a larger number of pregnant women may help in the understanding of the conjunctivitis seasonality variation in this group. The adequate documentation of data in the EHR is detrimental for the development of large retrospective studies. Possible ICD-10 code misclassifications might also have negatively impacted our findings. Approximately 80% of the data generated is unstructured, which turns the analysis more difficult. The data warehouse enables the analysis of different sources of information, such as numbers, texts and tables. However, this program has limitations regarding unstructured data, such as images and graphics. For this reason, it is important to have ophthalmologists involved in the development process.^(29)^ A prospective population- based observational study design would help to address the study limitations and provide stronger evidence to be used as comparison to the other studies.

The use of the data warehouse has an important implication for practice which is the possibility of near real-time data feeds that will ultimately bring benefits to patient care at an individual level, and also for public health strategies. It is also possible to include real-time scans for population screening requirements. The data warehouse could be used to provide knowledge to measure, and improve patient outcomes. The big data contained in the data warehouse can also be used to bring up facts that could not be perceived otherwise. One of the greatest assets of this type of database is that, not only does it can help provide answers to many outcome questions, but it can also assist in developing prospective trials and studies in which targeted data is required. It is a useful technology for data-intensive institutions, which are growing larger and more complex while the data cannot be processed by traditional applications.

## CONCLUSION

This retrospective epidemiological study using the data warehouse obtained similar results to previous epidemiological studies using other method of acquiring the data. The data warehouse is a useful methodology for epidemiological studies involving large number of patients, as well as an important tool in data management and analysis in the big data era. The main ophthalmic conditions observed in pregnant women during this study were dry eye, conjunctivitis, and ametropia. Conjunctivitis in this group did not present the same seasonal pattern as the general population of patients seen in same period at the same hospital. The mean intraocular pressure of pregnant women was higher in the first trimester. Data on the main ophthalmic manifestations during pregnancy could be useful for the development of public health strategies.

## References

[1] Alves M, Forschini RA, Schor P. Using Big Data in eye research to answer important scientific questions. Arq Bras Oftalmol. 2019;82(3):V-VI. 10.5935/0004-2749.20190044 31116304

[2] Schork NJ. The big data revolution and human genetics. Hum Mol Genet. 2018;27(R1):R1. 10.1093/hmg/ddy123 29672687

[3] Kortüm K, Hirneiß C, Müller M, Babenko A, Kampik A, Kreutzer TC. The influence of a specific ophthalmological electronic health record on ICD-10 coding. BMC Med Inform Decis Mak. 2016;16:100. 10.1186/s12911-016-0340-1 PMC4962360 27460682

[4] Schneeweiss S, Avorn J. A review of uses of health care utilization databases for epidemiologic research on therapeutics. J Clin Epidemiol. 2005;58(4):323-37. Review. 10.1016/j.jclinepi.2004.10.012 15862718

[5] Balyen L, Peto T. Promising artificial intelligence-machine learning-deep learning algorithms in Ophthalmology. Asia Pac J Ophthalmol (Phila). 2019; 8(3):264-72. Review. 10.22608/APO.2018479 31149787

[6] Naderan M, Jahanrad A. Topographic, tomographic and biomechanical corneal changes during pregnancy in patients with keratoconus: a cohort study. Acta Ophthalmol. 2017;95(4):e291-e6. 10.1111/aos.13296 27781383

[7] Naderan M. Ocular changes during pregnancy. J Curr Ophthalmol. 2018; 30(3):202-10. Review. 10.1016/j.joco.2017.11.012 PMC6127369 30197948

[8] Quigley HA, Broman AT. The number of people with glaucoma worldwide in 2010 and 2020. Br J Ophthalmol. 2006;90(3):262-7. 10.1136/bjo.2005.081224 PMC1856963 16488940

[9] Efe YK, Ugurbas SC, Alpay A, Ugurbas SH. The course of corneal and intraocular pressure changes during pregnancy. Can J Ophthalmol. 2012; 47(2):150-4. 10.1016/j.jcjo.2012.01.004 22560420

[10] Green K, Phillips CI, Cheeks L, Slagle T. Aqueous humor flow rate and intraocular pressure during and after pregnancy. Ophthalmic Res. 1988;20(6): 353-7. 10.1159/000266751 3237393

[11] Qureshi IA. Measurements of intraocular pressure throughout the pregnancy in Pakistani women. Chin Med Sci J. 1997;12(1):53-6. 11243101

[12] Ebeigbe JA, Ebeigbe PN, Ighoroje A. Ocular changes in pregnant Nigerian women. Niger J Clin Pract. 2012;15(3):298-301. 10.4103/1119-3077.100624 22960964

[13] Samra KA. The eye and visual system in pregnancy, what to expect? An in-depth review. Oman J Ophthalmol. 2013;6(2):87-91. Review. 10.4103/0974-620X.116626 PMC3779421 24082665

[14] Base de Dados Portugal Contemporâneo (PORTADA). Retrato de Portugal: indicadores 2011. Paris: PORDATA [citado 2019 Dez 30]. Disponível em: https://www.pordata.pt/Europa/Idade+m%c3%a9dia+da+m%c3%a3e+ao+nascimento+de+um+filho-2408

[15] Brauner SC, Chen TC, Hutchinson BT, Chang MA, Pasquale LR, Grosskreutz CL. The course of glaucoma during pregnancy: a retrospective case series. Arch Ophthalmol. 2006;124(8):1089-94. 10.1001/archopht.124.8.1089 16908810

[16] Goldich Y, Cooper M, Barkana Y, Tovbin J, Lee Ovadia K, Avni I, et al. Ocular anterior segment changes in pregnancy. J Cataract Refract Surg. 2014;40(11):1868-71. 10.1016/j.jcrs.2014.02.042 25217070

[17] Akar Y, Yucel I, Akar ME, Zorlu G, Ari ES. Effect of pregnancy onintraobserver and intertechnique agreement in intraocular pressure measurements. Ophthalmologica. 2005;219(1):36-42. 10.1159/000081781 15627826

[18] Martins TG, Martins TG. Ophthalmologic manifestations in pregnancy. Insight (San Fran.). 2020;45(1):23.

[19] Wegmann TG, Lin H, Guilbert L, Mosmann TR. Bidirectional cytokine interactions in the maternal-fetal relationship: is successful pregnancy a TH2 phenomenon? Immunol Today. 1993;14(7):353-6. Review. 10.1016/0167-5699(93)90235-D 8363725

[20] Gotovac M, Kaštelan S, Lukenda A. Eye and pregnancy. Coll Antropol. 2013; 37(Suppl 1):189-93. 23837242

[21] Loukovaara S, Harju M, Kaaja RJ, Immonen IJ. Topographic change in the central macula coupled with contrast sensitivity loss in diabetic pregnancy. Graefes Arch Clin Exp Ophthalmol. 2003;241(8):607-14. 10.1007/s00417-003-0692-y 12883910

[22] Manges TD, Banaitis DA, Roth N, Yolton RL. Changes in optometric findings during pregnancy. Am J Optom Physiol Opt. 1987;64(3):159-66. 10.1097/00006324-198703000-00001 3578480

[23] Sethi HS, Naik M, Gupta VS. Management of glaucoma in pregnancy: risks or choices, a dilemma? Int J Ophthalmol. 2016;9(11):1684-90. Review. 10.18240/ijo.2016.11.24 PMC5145101 27990376

[24] Mackensen F, Paulus WE, Max R, Ness T. Ocular changes during pregnancy. Dtsch Arztebl Int. 2014;111(33-34):567-75; quiz 576. Review. 10.3238/arztebl.2014.0567 PMC4165189 25220071

[25] Vigil-De Gracia P, Ortega-Paz L. Retinal detachment in association with pre-eclampsia, eclampsia, and HELLP syndrome. Int J Gynaecol Obstet. 2011;114(3):223-5. Review. 10.1016/j.ijgo.2011.04.003 21719013

[26] Errera MH, Kohly RP, da Cruz L. Pregnancy-associated retinal diseases and their management. Surv Ophthalmol. 2013;58(2):127-42. Review. 10.1016/j.survophthal.2012.08.001 23410822

[27] De Silva SR, Riaz Y, Watson SL. Monitoring diabetic retinopathy in pregnancy: meeting the NICE guidelines. Acta Ophthalmol. 2012;90(3):e243-4. 10.1111/j.1755-3768.2011.02158.x 21518305

[28] Rohr Thomsen C, Brink Henriksen T, Uldbjerg N, Milidou I. Seasonal variation in the hypertensive disorders of pregnancy in Denmark. Acta Obstet Gynecol Scand. 2020;99(5):623-30. 10.1111/aogs.13786 32020602

[29] Turano A, Eibling D. Using voogle to search within patient records in the VA corporate data warehouse. Fed Pract. 2019;36(11):518-23. PMC6913608 31892775

